# Transient ice loss in the Patagonia Icefields during the 2015–2016 El Niño event

**DOI:** 10.1038/s41598-022-13252-8

**Published:** 2022-06-10

**Authors:** Demián D. Gómez, Michael G. Bevis, Robert Smalley, Michael Durand, Michael J. Willis, Dana J. Caccamise, Eric Kendrick, Pedro Skvarca, Franco S. Sobrero, Héctor Parra, Gino Casassa

**Affiliations:** 1 Division of Geodetic Science, School of Earth Sciences, The Ohio State University, Columbus, OH USA; 2 Center for Earthquake Research and Information, The University of Memphis, Memphis, TN USA; 3 Department of Geological Sciences, University of Colorado, Boulder, CO USA; 4 National Geodetic Survey, National Oceanic and Atmospheric Administration, Silver Spring, MD USA; 5 Glaciarium, El Calafate, Santa Cruz Argentina; 6 Instituto Geográfico Militar, Santiago de Chile, Chile; 7 Glaciology and Snow Unit, General, Water Directorate, Ministry of Public Works, Santiago de Chile, Chile

**Keywords:** Cryospheric science, Climate change, Climate-change impacts, Cryospheric science

## Abstract

The Patagonia Icefields (PIF) are the largest non-polar ice mass in the southern hemisphere. The icefields cover an area of approximately 16,500 km^2^ and are divided into the northern and southern icefields, which are ~ 4000 km^2^ and ~ 12,500 km^2^, respectively. While both icefields have been losing mass rapidly, their responsiveness to various climate drivers, such as the El Niño-Southern Oscillation, is not well understood. Using the elastic response of the earth to loading changes and continuous GPS data we separated and estimated ice mass changes observed during the strong El Niño that started in 2015 from the complex hydrological interactions occurring around the PIF. During this single event, our mass balance estimates show that the northern icefield lost ~ 28 Gt of mass while the southern icefield lost ~ 12 Gt. This is the largest ice loss event in the PIF observed to date using geodetic data.

## Introduction

The climate in South America is strongly affected by the El Niño-Southern Oscillation (ENSO), with El Niño (EN) being ENSO’s negative phase. ENSO events are produced by an unstable interaction between the Pacific Ocean and the atmosphere in the equatorial zone^1^. The ENSO quasi-cycle has positive, neutral, and negative phases, with one whole ‘cycle’ typically lasting about 4 years. The positive and negative phases correlate with rainfall and temperature anomalies, and changes in windfields, cloud cover and solar insolation that generate large deviations from average seasonal weather patterns. The negative phase of ENSO, EN, for instance, correlates with negative precipitation and positive temperature anomalies in Southern Patagonia^2–5^. Inter-annual changes in weather patterns can have a significant impact on seasonally-adjusted ice loss, driving both positive and negative accelerations in the rate of change of ice mass^6^. Long-term climate change has slowly but relentlessly increased global oceanic and atmospheric temperatures, making it easier for atmospheric and oceanic quasi-cycles, such as the North Atlantic Oscillation (NAO), the Pacific Decadal Oscillation (PDO), or the ENSO to push local conditions beyond thresholds for major increases in ice loss. Approaching or crossing such thresholds was not possible even as little as 3 decades ago^7^.

There is strong evidence that the ENSO has responded to human activity, specifically greenhouse gas emissions, since the early 1990’s^8^. ENSO events are tending to strengthen with each new cycle, as manifested by the Oceanic Niño Index (see Supplementary Fig. S1). Studies from Li et al.^2^ and Galván et al.^9^ in South America show the 2015–2016 EN event exhibited large temperature and precipitation anomalies that generated visible loading signals in geodetic time series. Studies from Lo Vecchio et al.^10^ and Willis et al.^11^ have quantified both the long-term trend and variable inter-annual ice loss in the Patagonia Icefields (PIF) as well as ice loss for specific outlet glaciers that may be influenced by the intensification of EN events. To date, no studies have reported the PIF ice loss during a specific EN event, mostly due to a lack of observations and/or the low temporal resolution of the available datasets. Due to the paucity of on-site observations, most previous studies are based on remote sensing data from satellite images^10–12^, and time-variable gravity data from the Gravity Recovery and Climate Experiment (GRACE) and GRACE Follow-On (GRACE-FO).

Satellite images and GRACE present some challenges when attempting to develop models with dense temporal and spatial resolution. For the case of visible satellite images, cloud coverage in Patagonia is frequent, limiting the number of usable images available for analysis. Other observational methods, such as synthetic aperture radar (SAR), penetrate cloud cover (e.g.^13^). Both SAR and visible images, however, need to be paired with other observation techniques, such as GRACE and GPS, to correctly constrain mass changes. For time-variable gravity data from GRACE and GRACE-FO, the inherent spatial resolution of ~ 330 km is too coarse to independently estimate the ice loss at the scale of the internal drainage basins in the PIF. Also, due to the discontinuity between GRACE and GRACE-FO missions, there is a data gap of ~ 12 months, during part of the ENSO cycle between 2017 and 2018. Although this gap has been successfully filled with other models^14^, it should be noted that the later GRACE time series are noisier due to strategies to extend the mission.

Other studies analyzed long-term trends using campaign GPS data, measured every 1–2 years, to understand glacial isostatic adjustment (GIA) and short-term elastic response signals^15,16^. These studies, however, are limited by temporal aliasing due to undersampling of the annual and semi-annual signals observed in GPS time series. For 2-year campaign intervals the Nyquist period is 4 years, roughly the same as the mean ENSO period. Continuous GPS (CGPS) data are therefore required to fully capture the crustal displacements due to short-term oscillations and trends in ice mass.

CGPS data provide higher spatiotemporal resolution (relative to campaign data) to constrain the PIF ice loss during the 2015–2016 EN event. Although the density of the CGPS data in the region is low, the addition of the CGPS ground-based data with satellite data facilitates estimating the episodic or transient ice loss during the 2015–2016 EN. Using an 8-year time series of daily positions from seven CGPS stations, an elastic response model^17^, stage data from all major lakes in southern Patagonia, SRTM data, ice velocity from satellite images^18^, glacier surface height change grids^19^, and the Global Land Data Assimilation System^20^ (GLDAS) terrestrial water storage (TWS) model, we produced a least-squares ice loss estimate for the North and South PIF. Our results show a significant acceleration in ice loss during the first months of 2016 in agreement with previous studies^2^. Our study, however, provides better spatial resolution that allows separating the northern and southern PIF ice loss signals. Thus, our results show an increased ice loss in the northern PIF compared to previous estimates of ice loss rates. Our analysis supports the hypothesis that this increased ice loss rate may have been driven by the 2015–2016 EN event, and the mass loss is likely permanent as the ice mass had not recovered by late 2018, the end of our study period.

## GPS observations in the Patagonia Icefields

The elastic response of earth’s crust due to loading by large hydrological cycles, which are rarely steady or strictly periodic, is well established (e.g.^21^). A major driver of crustal loading are changes in TWS. Galván et al.^9^ has shown the effects of the 2015–2016 EN in the Amazon basin and Li et al.^2^ used GRACE to constrain the fluctuations in PIF inter-annual ice loss during the 2015–2016 EN event. GRACE studies of the PIF^2^, however, reported accelerating ice loss at the beginning of 2016 without isolating the ice loss during the 2015–2016 EN. Additionally, the coarse resolution of GRACE does not permit direct observation of the separate Northern (NPIF) and Southern PIF (SPIF) ice mass losses.

**Figure 1:**
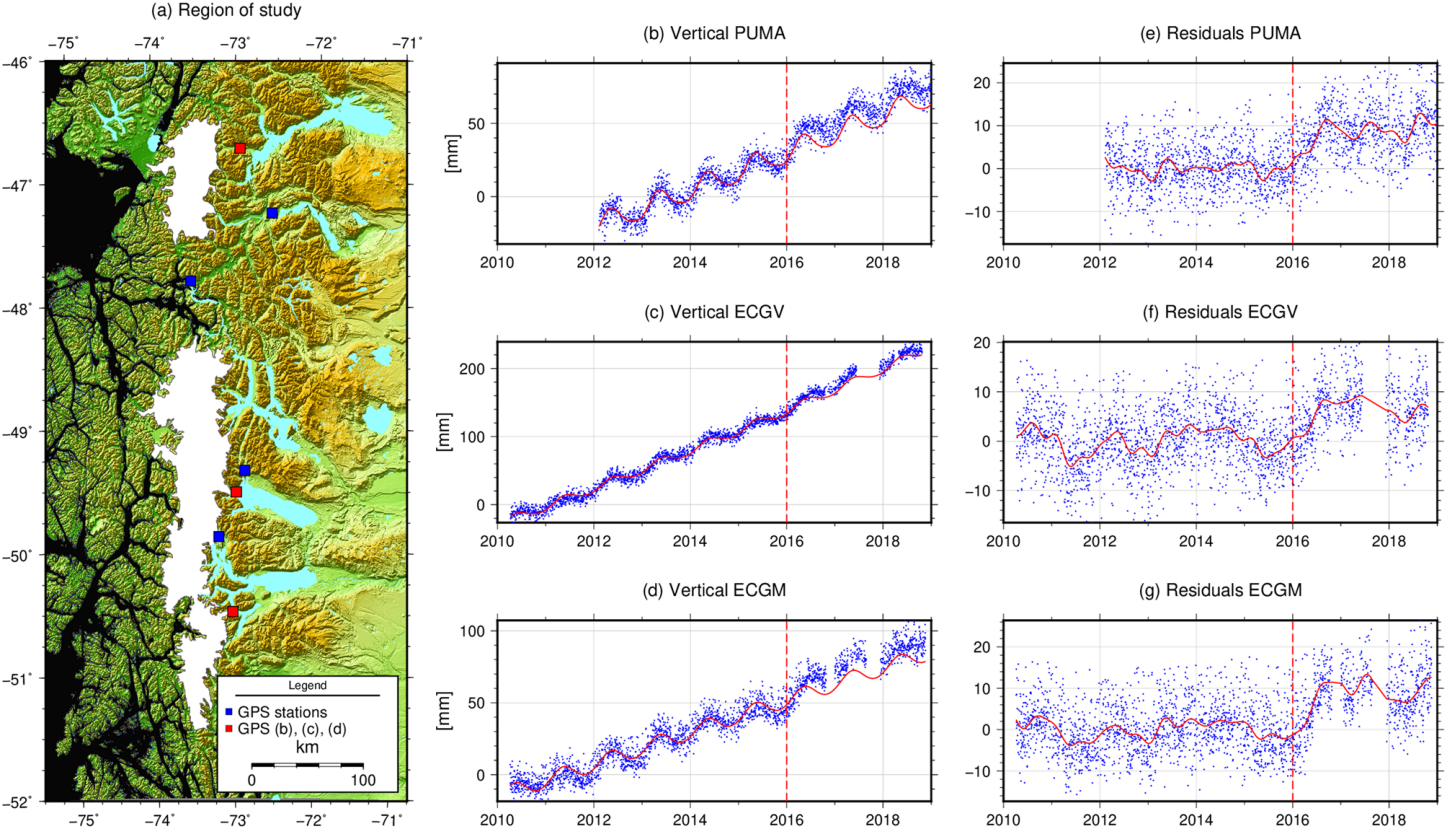
(**a**) Region of the Northern and Southern Patagonia Icefields (map created using the Generic Mapping Tools^37^ version 6, https://docs.generic-mapping-tools.org/6.0/) showing the location of the CGPS stations used in this study. Stations, north to south: PUMA, COCR, TRTL, CHLT, ECGV, ECGU, ECGM. (**b**–**d**) North to south, vertical component of GPS stations PUMA, ECGV, ECGM; blue dots are daily solutions obtained with GAMIT-GLOBK; red curves represent the trajectory model fit for data before 2016; trajectory fit goes from start of each time series to the vertical red dashed lines; (**e**–**g**) residuals after removal of the trajectory model fits; red curves represent a least-squares collocation smoothed signal of the residuals to reduce noise in the GPS solutions.

To obtain the spatiotemporal resolution necessary to quantify the ice loss during the 2015–2016 EN, one requires continuous in-situ observations such as CGPS data. Figure 1a shows the seven PIF CGPS stations used in this study from the Chilean and Argentinian continuous GNSS networks^22,23^. Figure 1b–d show the vertical components from three of the seven CGPS stations processed using GAMIT-GLOBK^24^. The GPS data processing scheme used the latest orbits and antenna calibration parameters available from the International GNSS Service, the Vienna Mapping Functions^25^ to estimate the atmospheric delays, atmospheric tidal and non-tidal corrections, and the ocean tide loading model FES2014b^26^. Our time series show the evolution of the CGPS trajectories in the IGS14 reference frame realized using a regional densification with over 400 stations in Central and South America, the Caribbean, and Antarctica^27^.

We fit parametric trajectory models to each CGPS station composed of a linear trend, annual and semi-annual seasonal components, and, whenever possible (i.e. when more than 4 years of data are available), a quadratic term^28^. The linear and quadratic components remove reference frame, long-term ice loss, GIA, and any slow but steady effects of acceleration on ice loss. The periodic components remove seasonal variations including reference frame-related, local geodynamic effects, and any processing artifacts. To avoid any influence from possible EN effects in the trajectory parameters we only fit data before 2016. The residuals on Fig. 1e–g, therefore, show departures from the nominal station behavior recorded over the previous 6 years. To separate the EN effect from noise in the GPS time series, we applied a non-parametric least-squares collocation smoothing filter^29,30^.

Figure 1e–g show a clear offset of ~ 10 to 12 mm after 2016 with respect to the trajectory model. This shows that during 2016 there was a significant change in the vertical component behavior. While loading also produces smaller horizontal signals, we do not consider them due to the low signal-to-noise ratio of these observations. We will explain the observed time series behavior in terms of an ice loss unloading event that created the vertical displacement transient observed in the CGPS time series.

## Methods

We model the observed vertical displacement change (obtained after removing any reference frame components, long-term ice loss, GIA, etc.) at each CGPS site as

1 $$d_{U} = d_{GLDAS} + d_{Lakes} + d_{Ice}$$

where $d_{U}$ is the observed vertical displacement change at the CGPS station, $d_{GLDAS}$ is the TWS loading vertical displacement, $d_{Lakes}$ is the lake stage-induced loading vertical displacement, and $d_{Ice}$ is the loading vertical displacement due to non-steady and non-seasonal ice changes. Therefore, the $d_{GLDAS}$ and $d_{Lakes}$ components are also detrended using the same parametric trajectory model used to detrend $d_{U}$ (linear, seasonal, etc.). Using the 0.25 × 0.25 degree GLDAS Catchment Land Surface Model (version 2.0) TWS, PREM^31^, and the elastic response to a disk load (using 5 km diameter disks), we computed the vertical displacement of the crust produced by the regional changes in TWS at the seven CGPS stations between 2010 and 2019. Because GLDAS does not correctly account for ice fields and water bodies, we removed the model cells within these areas. Using the same elastic disk load and elastic models, we computed the water storage change signal for the lakes in Patagonia using lake stage observations (obtained from the Argentine National System of Hydrological Information, see http://bdhi.hidricosargentina.gob.ar/). Thus, the ice load signal can be found from Eq. (1) as

2 $$d_{Ice} = d_{U} - \left( {d_{GLDAS} + d_{Lakes} } \right)$$

**Figure 2:**
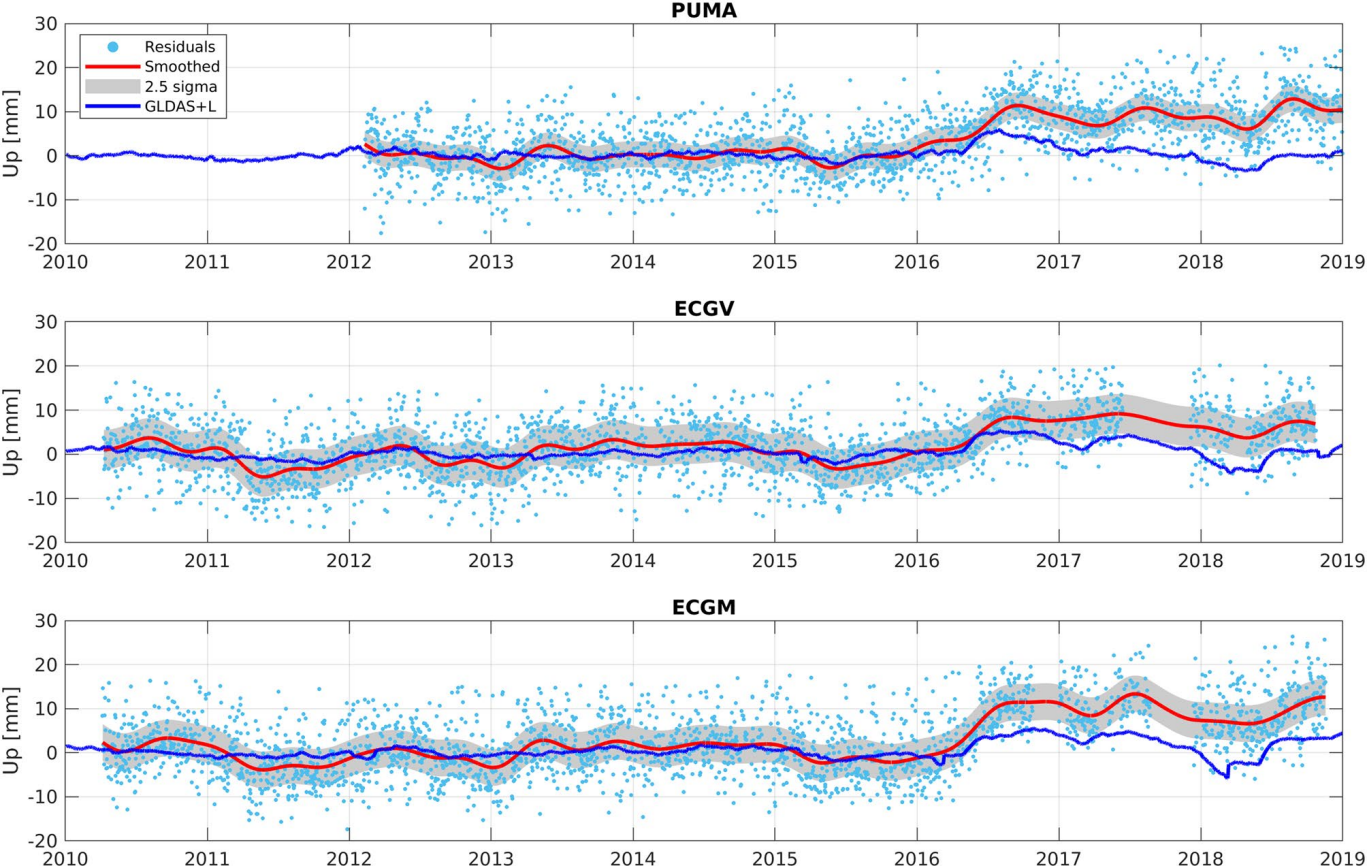
Vertical residuals for CGPS PUMA, ECGV, and ECGM including ground elastic response from the GLDAS + L model. A clear offset is visible after March 2016 when the CGPS time series show an additional uplift relative to the GLDAS + L model.

The $d_{GLDAS}$+$d_{Lakes}$ time series residuals (hereafter, GLDAS + L) in Fig. 2 also show departures from the nominal GLDAS + L behavior estimated between 2010 and 2016. Figure 2 shows the same time series as in Fig. 1e–g together with the detrended GLDAS + L time series. Before 2016, both time series show the same approximate behavior with no clear offset between them. Nevertheless, a clear offset of ~ 5–8 mm is visible between the GLDAS + L and the CGPS residual time series after 2016. In some cases, offsets of ~ 10 mm can be observed, especially after 2018. These offsets are above the observed standard deviation between the smoothed CGPS signals and the GLDAS + L signal, which is ~  ± 2 mm (Fig. 2, shaded area shows the 2.5 sigma region).

As previously discussed, the GLDAS + L time series do not include any loading effects due to changes in ice mass. Thus, the ice loss can be estimated from Eq. (2), hereafter CGPS-GLDAS-L. We discretized the NPIF and SPIF using 1 km diameter disks to find the ice loading signal using the CGPS data. To account for the spatial variation of ice loss, the elastic response of the disks (at each CGPS station) was calculated using the rate of change of the ice surface height (dh/dt) grids for NPIF and SPIF from Braun et al.^19^. Although the spatial distribution of dh/dt rates in the PIF during the 2015–2016 EN are likely to show differences relative to the average dh/dt rates reported by Braun et al., we will use this pattern as a more reasonable estimate of the spatial variation for ice loss during the 2015–2016 El Niño than a uniform one, and scale it to match the CGPS data. In either case, the total ice mass loss is controlled by the CGPS data, not the ice mass loss distribution. The elastic response of each disk-station pair can be converted from rate, dh/dt, to a height change by multiplying the rate by a time interval, Δt. It should be noted that if one knows the ice height change, the disk loading least-squares problem can be interpreted as a solution for the density of the disks, rather than the equivalent water height, that generates the observed CGPS displacements. The dh/dt grids, however, represent the average ice height change in the data used by Braun et al., and these values are not representative of the ice loss during the 2015–2016 EN. We can assume, however, that scaling the dh/dt rates by a factor (one for NPIF and another for SPIF) will bring them into agreement with the ice loss during this EN event. In other words, we wish to transform the average dh/dt values into an ‘instantaneous’ ice loss that fits the CGPS data during the 2015–2016 EN. Therefore, the loading problem presented here consists of estimating the appropriate Δt scaling factor for each field such that the densities estimated from the elastic response (at the CGPS stations) are within the known range of ice density. This Δt scaling factor gives the ratio of ice loss rate during 2015–2016 EN with respect to that obtained by Braun et al.

The mass balance problem was solved using three distinct approaches to characterizing ice density variation in the PIF. This diversity of approach helps us to assess the robustness of our conclusions. In the first approach, hereafter model A, we assume that density is a linear function of height, assuming an ice density of 918 and 500 kg/m^3^ at the lowest and highest elevations of the PIF, respectively. The second and third approach, models B and C, categorized the disks into two and three groups, respectively, based on elevation, ice velocity, and dh/dt values. The elevation of each disk was obtained using 30 m SRTM data, and the ice velocities were obtained from the NASA Regional Glacier and Ice Sheet Surface Velocities retrieved from the ITS_LIVE project^18^.

The classification into groups was determined using a height threshold (HT, varying from ~ 1000 to 1300 m) following Bravo et al.^32^ and Schaefer et al.^33^, a velocity threshold (VT, set at 30 cm/day), and the dh/dt value for each disk (negative/positive for height loss/gain). Disks were classified as group 1 (G1, lowest density) when dh/dt > 0 (except when velocity > VT, e.g. on Glaciar Pío XI, see^13^), as group 2 (G2, mid density) when dh/dt < 0, and velocity < VT or height > HT, and group 3 (G3, highest density) when height < HT and dh/dt < 0. We combined G1 and G2 to simplify the problem (model B) and we used the three groups for model C. For all three models, we solved for disk densities using the average CGPS-GLDAS-L difference after 2016.5. Observations were weighed in the least-squares adjustment using the squared inverse of each CGPS-GLDAS-L standard deviation calculated using data before 2016.

To obtain the three pairs of Δt for the NPIF and SPIF dh/dt grids (one for each model), we performed a grid search over Δt and selected those that minimized the root mean square (rms) misfit between estimated and a priori density parameters (Supplementary Fig. S2). No constraints were applied to the least squares problems, except for model C where we applied a soft constraint requiring the density of G2 to be the average of G1 and G3.

## Results

**Figure 3:**
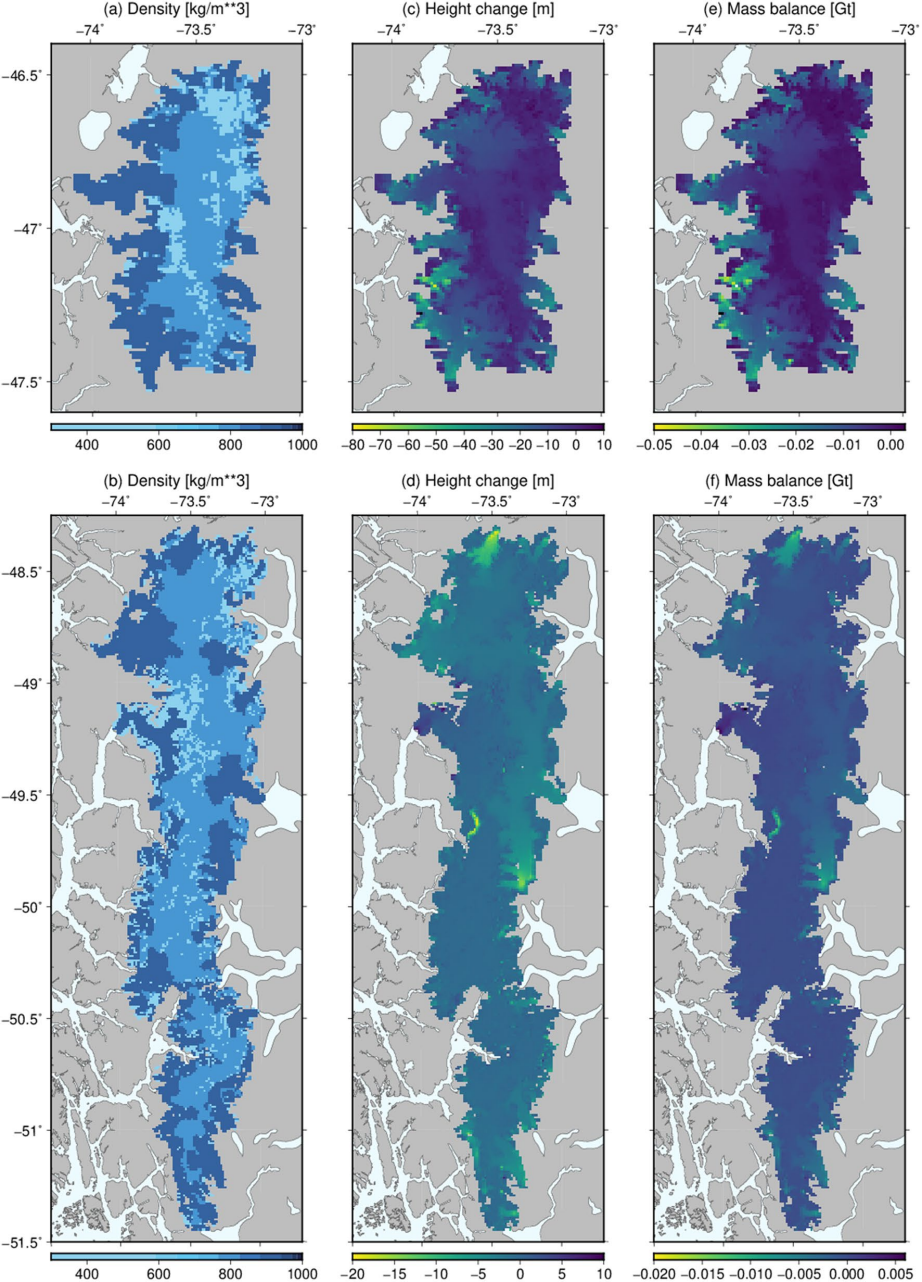
Results for model C (maps created using the Generic Mapping Tools^37^ version 6, https://docs.generic-mapping-tools.org/6.0/). Disk densities for NPIF (**a**) and SPIF (**b**). Height changes after multiplying the dh/dt grids by Δt for NPIF (**c**) and SPIF (**d**). Mass balance grid for NPIF (**e**) and SPIF (**f**) using the estimated densities from (**a**) and (**b**) and the height change from (**c**) and (**d**).

Table 1 shows the results for each model, A, B, and C, including the density parameters. As in model A, models B and C required an a priori density to compute the rms misfit during the grid search. While model B used the same density range as A, 918 and 500 kg/m^3^ for G3 and G1 + G2, respectively, model C used 500, 740, and 918 kg/m^3^ for G1, G2, and G3 respectively. Results for model C are shown in Fig. 3a, b, where we note that the lowest density estimation (G1) was ~ 400 kg/m^3^.

**Table 1:** Δt values, model results for density as a function of height (h) (A), a two-disk type classification (B), and a three-disk classification (C), NPIF and SPIF mass change and model RMS misfit.

Models	NPIF Δt (year)	SPIF Δt (year)	Ice densities (kg/m^3^)	NPIF mass change (Gt)	SPIF mass change (Gt)	RMS misfit (mm)
(A) δ = b + m × h	8.03	0.74	b: 925; m: − 0.1152	− 30.5	− 8.2	2.61
(B) G1 + G2, G3	7.60	0.97	G1 + G2: 497; G3: 923	− 29.1	− 10.9	2.10
(C) G1, G2, G3	6.90	0.95	G1: 397; G2: 767; G3: 915	− 28.2	− 11.7	2.03

The range of Δt for the three models does not show significant changes, and it is readily visible that the NPIF Δt is between 7 and 8 times the SPIF Δt value. This shows that the NPIF and SPIF dh/dt relationship for the 2015–2016 EN event is different from that obtained by Braun et al., which used SAR interferometry data over the years 2000 to 2011/2015. This deviation from the mean dh/dt behavior has been identified in previous studies (e.g.^2^) and is likely the result of specific climate dynamics related to the strong EN event. Other sources that can explain part of the deviation from the mean dh/dt also include contributions below lake or sea level which are not captured by the dh/dt models^19^. Figure 3c, d show the resulting height change for NPIF and SPIF, respectively. Some of the disks in NPIF show large height changes, reaching − 70 m, which are a direct result from the scaling of the dh/dt grid. Although these values may be considered outliers (due to the mismatch between the dh/dt values from Braun et al. and those from the time period of our study), we have not removed these disks from our results. The resulting mass change for each disk for NPIF and SPIF, obtained from the disk density inversion using the CGPS-GLDAS-L difference, is shown in Fig. 3e, f.

The rms misfit between the observed and modeled CGPS-GLDAS-L displacements of model B shows an improvement, from 2.61 mm for A, to 2.10 mm. These results show that for the same number of model parameters, the disk classification in model B performs better than a linear height-dependent disk density, which is not surprising. As expected, the rms misfit is further improved in model C when three families of disks are used. Although our models explain most of the CGPS-GLDAS-L displacements, the model appears to underestimate part of the vertical signal at the Perito Moreno glacier CGPS station (ECGM, see Supplementary Fig. S3).

We evaluated the uncertainty in the models by perturbing its input parameters. We tested the model sensitivity to the uncertainties of disk classification threshold values (i.e., some disks can change between groups based on the thresholds) and we found that the overall effect is negligible. We also perturbed the a priori ice densities by 7% to test the uncertainty in the mass balance solution and found that the changes in ice loss were always below 1% (0.1–0.2 Gt). This is in part because any misfit in the a priori ice densities will be corrected by the scale factor applied to the dh/dt grids. Thus, the most significant effect in the mass balance estimates is from the relative uncertainty between the GLDAS + L model and the CGPS data. We therefore perturbed the CGPS-GLDAS-L values using their standard deviations and found that for model C, the NPIF ice loss uncertainty is ± 8 Gt while the SPIF uncertainty is ± 2 Gt.

## Discussion

A strong link between ENSO and surface mass balance has not been demonstrated in Patagonia, although Weidemann et al.^4^ showed that strong modes of climate variability produce large temperature, wind, and precipitation anomalies in Southern Patagonia. It should also be noted that the 2015–2016 EN correlated with the largest temperature and precipitation anomaly in the region since 2003^2^, which may have pushed local conditions beyond thresholds for major increases in ice loss.

After removing modeled reference frame, GIA, and periodic effects from the time series, we found the total mass loss for the PIF to be ~ 40 Gt ± 8.2 Gt. Our results are in agreement with previous studies, showing a dramatic ice loss in the first few months of 2016. Yet, our total ice loss estimate is approximately half of the mass loss reported by Li et al.^2^ during the same period (after correcting for the linear trend between 2013 and 2016 reported by the authors), which used GRACE data and the GLDAS model. We speculate that GRACE data noise may have introduced a bias in the results from Li et al. Results from Ciraci et al.^14^ (Supporting Information, Figure S3) also show a clear offset in the PIF mass loss trends, although the authors did not discuss it.

While previous studies attribute the largest ice loss signal to SPIF (e.g.^13,19^), our mass balance results attribute 75% of the ice loss to NPIF, which lost an equivalent of ~ 7 years of ice, while SPIF only lost ~ 1 year, at the average rate in the previous decade reported by Braun et al. We hypothesize that the location of NPIF relative to SPIF (further north) may have favored the NPIF ice loss compared to SPIF, which is supported by previous studies showing that accumulation in NPIF has decreased more significantly than at SPIF^7^.

This work shows, for the first time, the PIF ice loss during the 2015–2016 El Niño event using ground-based CGPS data. Our method combined direct observations of crustal deformation and remote sensing datasets to produce a robust mass balance estimate that has low sensitivity to variations in the a priori ice densities. Although the continuous geodetic data in Patagonia is sparse, the available dataset of seven stations allowed us to obtain ice loss estimates for both ice fields for this specific EN event. Our results suggest that the 2015–2016 EN had a large impact on the PIF, and highlights the necessity to further study the effects from strong modes of climate variability using ground-based GNSS/GPS data. Additionally, surface mass balance estimates from models such as MAR^34^, RACMO2^35^, and MERRA-2^36^ could provide means to extrapolate the ice loss time series to before the deployment of the CGPS network to investigate the sensitivity of the PIF to prior EN events.

## Supplementary Information

Supplementary Information.

## Data Availability

The continuous GNSS/GPS data is public and available through Instituto Geográfico Nacional de Argentina website https://www.ign.gob.ar/NuestrasActividades/Geodesia/Ramsac/DescargaRinex and the Centro Sismológico Nacional, Universidad de Chile website http://gps.csn.uchile.cl/. The lake stage data is available from the Argentine National System of Hydrological Information http://bdhi.hidricosargentina.gob.ar/. The GLDAS grids are available from https://disc.gsfc.nasa.gov/datasets?keywords=GLDAS. The Regional Glacier and Ice Sheet Surface Velocities are available from https://its-live.jpl.nasa.gov/#data.
