# Supplementary material for: Transient ice loss in the Patagonia Icefields during the 2015–2016 El Niño event
Source: Sci Rep. 2022 Jun 10;12:9553. doi: 10.1038/s41598-022-13252-8 (PMC9187772; doi:10.1038/s41598-022-13252-8)
Supplement: Supplementary file 1 — Supplementary Information. [file 41598_2022_13252_MOESM1_ESM.pdf]

Supplementary Information for
Transient Ice Loss in the Patagonia Icefields during the 2015-2016 El Niño Event

Demián D. Gómez[1], Michael G. Bevis[1], Robert Smalley, Jr.[2], Michael Durand[1], Michael J. Willis[3], Dana J. Caccamise II[4], Eric Kendrick[1], Pedro Skvarca[5], Franco S. Sobrero[1], Héctor Parra[6], Gino Casassa[7]

1. Division of Geodetic Science, School of Earth Sciences, The Ohio State University, Columbus, OH, USA
2. Center for Earthquake Research and Information, The University of Memphis, Memphis, TN, USA
3. Department of Geological Sciences, University of Colorado, Boulder, CO, USA
4. National Geodetic Survey, National Oceanic and Atmospheric Administration, USA
5. Glaciarium, El Calafate, Santa Cruz, Argentina
6. Instituto Geográfico Militar, Santiago de Chile, Chile (retired)
7. Glaciology and Snow Unit, General, Water Directorate, Ministry of Public Works, Chile

Contents of this file

Text S1
Table S1
Figures S1 to S3

S1. Details of the GNSS observations

Table S1 shows the locations of the continuous GPS stations from the *Red Argentina de Monitoreo Satelital Continuo*[1] and *Centro Sismológico Nacional*[2].

Table S1: List of GPS stations used for this study

Site ID	Location	Latitude [deg]	Longitude [deg]
PUMA	Valle de Leones, Aysén, Chile	-46.726081	-72.947252
CHLT	Chaltén, Santa Cruz, Argentina	-49.340456	-72.885562
COCR	Cochrane, Aysén, Chile	-47.252224	-72.562501
ECGM	Glaciar Perito Moreno, Santa Cruz, Argentina	-50.488252	-73.034965
ECGU	Glaciar Upsala, Santa Cruz, Argentina	-49.878703	-73.210061
ECGV	Glaciar Viedma, Santa Cruz, Argentina	-49.516640	-72.991842
TRTL	Tortel, Aysén, Chile	-47.803102	-73.541046

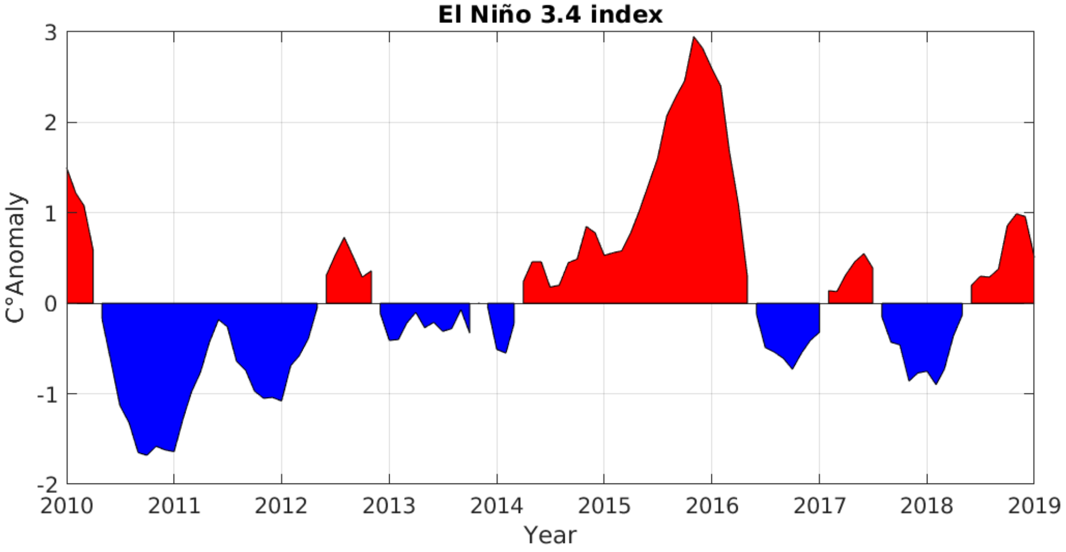

Fig. S1: Temperature anomalies from El Niño 3.4 index for the time period in this study. Largest positive anomaly clearly seen during the 2015-2016 El Niño event.

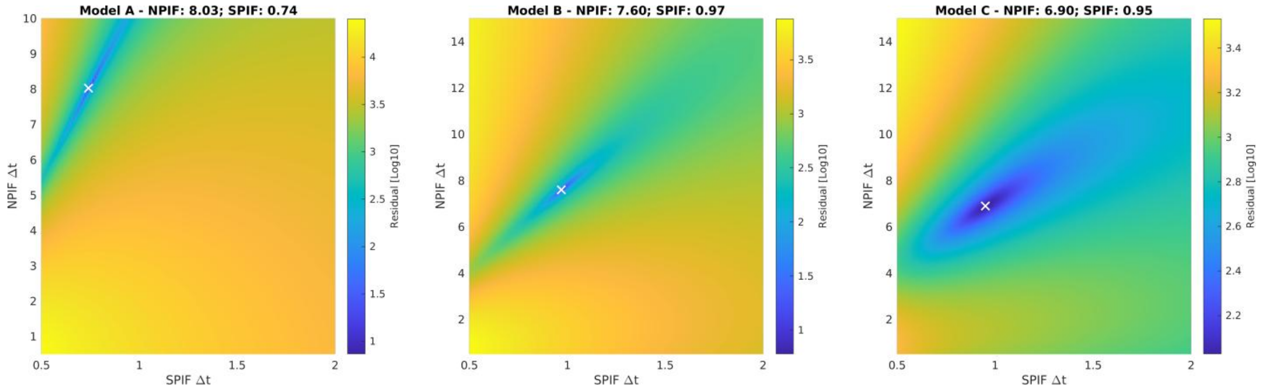

Fig. S2: Δt grid search for model A, B, and C. White cross shows the selected Δt pair that produces the minimum RMS misfit for the model densities.

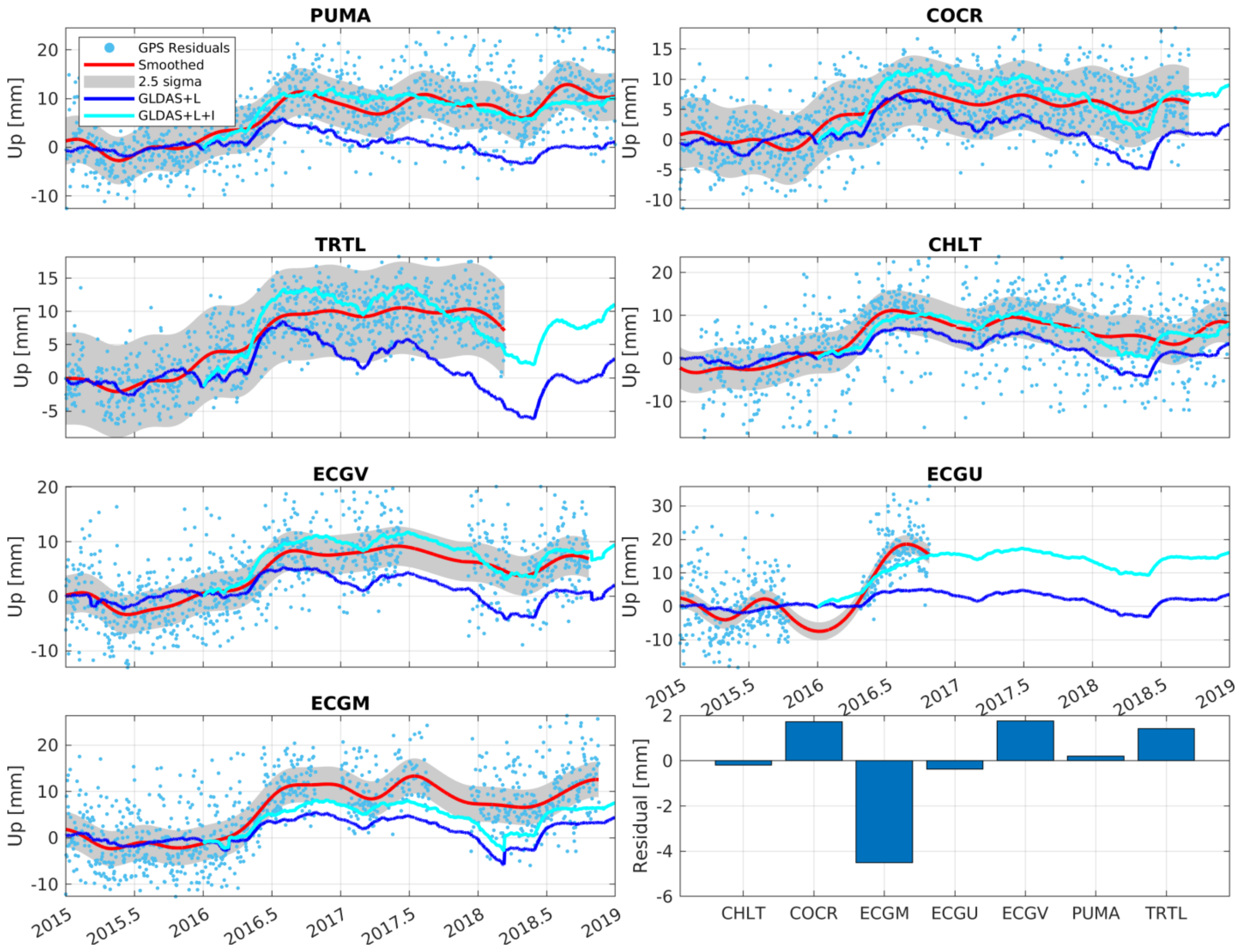

Fig S3: Vertical uplift signal including the ice loss unloading component (GLDAS+L+I) using the three disks model (C). Bottom right: misfit between the average CGPS vertical uplift and the average GLDAS+L+I modeled signal.

Data Sources

El Niño 3.4 index data was obtained from http://ftp.cpc.ncep.noaa.gov/wd52dg/data/indices

References

1. Piñón, D. A. *et al*. The History, State, and Future of the Argentine Continuous Satellite Monitoring Network and Its Contributions to Geodesy in Latin America. *Seismological Research Letters* **89**, 475–482 (2018).

2. Báez, J. C. *et al*. The Chilean GNSS Network: Current Status and Progress toward Early Warning Applications. *Seismological Research Letters* **89**, 1546–1554 (2018).
